# Defending against adversarial attacks on Covid-19 classifier: A denoiser-based approach

**DOI:** 10.1016/j.heliyon.2022.e11209

**Published:** 2022-10-22

**Authors:** Keshav Kansal, P Sai Krishna, Parshva B. Jain, Surya R, Prasad Honnavalli, Sivaraman Eswaran

**Affiliations:** a Research Center for Information Security, Forensics and Cyber Resilience, PES University, Bangalore, India; b Department of Electrical and Computer Engineering, Curtin University Malaysia, Miri, Sarawak, Malaysia

**Keywords:** Adversarial attacks, Denoiser, FGSM, PGD, HGD, Machine learning, Deep neural network

## Abstract

Covid-19 has posed a serious threat to the existence of the human race. Early detection of the virus is vital to effectively containing the virus and treating the patients. Profound testing methods such as the Real-time reverse transcription-polymerase chain reaction (RT-PCR) test and the Rapid Antigen Test (RAT) are being used for detection, but they have their limitations. The need for early detection has led researchers to explore other testing techniques. Deep Neural Network (DNN) models have shown high potential in medical image classification and various models have been built by researchers which exhibit high accuracy for the task of Covid-19 detection using chest X-ray images. However, it is proven that DNNs are inherently susceptible to adversarial inputs, which can compromise the results of the models. In this paper, the adversarial robustness of such Covid-19 classifiers is evaluated by performing common adversarial attacks, which include the Fast Gradient Sign Method (FGSM) and Projected Gradient Descent (PGD). Using these attacks, it is found that the accuracy of the models for Covid-19 samples decreases drastically. In the medical domain, adversarial training is the most widely explored technique to defend against adversarial attacks. However, using this technique requires replacing the original model and retraining it by including adversarial samples. Another defensive technique, High-Level Representation Guided Denoiser (HGD), overcomes this limitation by employing an adversarial filter which is also transferable across models. Moreover, the HGD architecture, being suitable for high-resolution images, makes it a good candidate for medical image applications. In this paper, the HGD architecture has been evaluated as a potential defensive technique for the task of medical image analysis. Experiments carried out show an increased accuracy of up to 82% in the white box setting. However, in the black box setting, the defense completely fails to defend against adversarial samples.

## Highlights

• The current methods of testing for the virus are RT-PCR and RAT. These test results may take up to 24 h. Recent studies and research have shown that lung images show the presence or damage caused by the virus using machine learning techniques.
• Adversarial Attack is a major security threat in the domain of machine learning. Adversarial training is the most widely explored technique to defend against adversarial attacks.
• High-level representation Guided Denoiser (HGD) architecture, another defensive technique, being suitable for high-resolution images, makes it a good candidate for medical image applications.
• HGD architecture has been evaluated as a potential defensive technique for the task of medical image analysis with a new loss function.
• In a White box scenario considerable increase in accuracy is seen. However, in the black box setting, the defense fails to defend against adversarial samples.

## 1 Introduction

Covid-19 [1], claimed to have origins in China, has been tormenting the world since early December 2019. The outbreak has been so severe that governments have had to impose strict travel bans and lockdowns to restrict the movement of individuals. Studies show that the virus takes anywhere between 7 and 14 days to mature within human bodies, and by this time, the infected individuals have already spread it to three others on average. The infection rate has been growing exponentially. The current methods of testing for the virus are RT-PCR [2] and RAT [3]. The accuracy of the tests is not well established, and results may take up to 24 h. There are several limitations to these methods as well. The availability of testing kits, qualified professionals to collect samples, and assessing large numbers of samples at labs are the main challenges. Recent studies and research have shown that lung images show the presence or damage caused by the virus. Chest X-Rays [4] turned out to be a good candidate for detection of the virus. Radiologists can examine chest X-rays and can speculate on the presence of the virus. The number of such qualified radiologists is scarce.

Researchers have been exploring the use of Machine Learning in detecting the virus [5, 6]. However, due to the unavailability of quality datasets, the studies are still in a nascent form. These studies have been relying on data manipulation techniques and augmentations to increase the sample size. A pertinent question that often arises when Machine Learning systems are introduced in the medical field [7] is about the risks involved in allowing machines to work autonomously in making decisions. Many autonomous robots are already in trials, and a major breakthrough is with the FDA-approved IDx-DR [8] to perform diabetic retinopathy. Such apprehensions are valid and highlight the need for more research in addressing such security concerns. Countries like China, Canada, Iran, and South Korea [9, 10] have already begun implementing such Machine Learning models as part of testing protocols in select labs. This paper explores the accuracy of such Machine Learning Models and attempts to improve their robustness against adversarial attacks like the Fast Gradient Sign Method and Projected Gradient Descent. A defensive technique, High-Level Representation Guided Denoiser, has been evaluated to defend against such attacks.

## 2 Literature survey

The identification of Covid-19 through chest X-rays is a relatively new problem, with few published works giving positive results. Studies have explored several architectures such as CNN, DarkNet, and Logistic Regression for the purpose of classification using chest X-ray and CT scan images. Countries like China, South Korea, and Canada have also been testing the use of such models in real-time labs. Most of the studies propose training multiclass classifiers since it is difficult to differentiate between pneumonia and Covid-19. One of the biggest problems highlighted by these studies is the dataset constraint. In some studies, this problem has been overcome with Transfer Learning, which yields better results on smaller datasets. The results have been documented in the survey paper [10].

Experiments performed in [11] indicate that medical images are more susceptible to adversarial noise due to the nature of the medical images and the Deep Neural Networks (DNNs) used for their classification. The authors explain that when compared to normal images, less noise is required to be added in the case of medical images for a successful adversarial attack. This is because the attention of the model shifts to regions that do not necessarily affect the classification. The paper also demonstrates that DNNs used for medical imaging tasks are highly vulnerable to adversarial samples due to overparameterization. This indicates that the threat of Adversarial Attacks in the case of medical imaging analysis is very high.

The adversarial samples generated for one target model are highly likely to fool any other model for the same application, irrespective of the model architecture [12]. This is because the models trained for a specific application learn similar functions, and hence an attacker can perform a successful adversarial attack without having direct access to the target model just by creating their own model for the same application.

Many different methods of creating adversarial samples are discussed by Ren et al. [13]. These include the FGSM attack, L-BFGS attack, Iterative attacks, and Saliency map methods. FGSM attack is simple, in a way that, rather than minimizing the loss by tuning the weights of the network during backpropagation, the attack tunes the input data to maximize the loss based on the same backpropagation gradients. In simpler words, the input is changed in such a way that the loss is maximum for the original class of the input.

The PGD attack, a white-box attack setup, is one of the most powerful first-order adversaries [14, 15]. Formulating the PGD attack in itself is an optimization problem. It attempts to find the perturbation that maximizes the loss of a model on a particular input while keeping the size of the perturbation small. The L^2^ or L∞ norm of the perturbation is added so that the content of the adversarial sample is the same as the clean sample. PGD generated samples show a decline in the accuracy of the classifier (from 87% to 46%). Adversarial training, a popular defense technique, yields an accuracy improvement of only 64%.

Adversarial training involves training the model with adversarial samples. This method has proved to highly increase the model accuracy. However, it leads to a problem of label leaking wherein the new model can classify adversarial samples correctly, but its accuracy decreases when classifying clean images [16], hence a gain in robustness at the cost of accuracy. High-Level Representation Guided Denoiser (HGD), a novel defense architecture, has been proven to be an effective defense against adversarial attacks [17]. Adversarial training takes a long time to train the model and is computationally very expensive. Its output on a new sample on which it was not trained is not authentic, i.e., it does not make the model completely robust. The denoisers are like the first steps taken towards making the model more robust.

Xiao et al. proposes a novel loss function that could be utilized by existing denoisers to perform better. The new loss function uses the reconstruction error at different higher-level representations of the image in the target model architecture and helps in reducing the residual error and guiding better training. The HGD aims to remove the high-level influence of adversarial noise. To test the novel loss function, the authors used a DUNET, a denoising architecture, and trained it using the new loss function. This paper uses HGD architecture in experiments to address the problem of adversarial attacks in the medical domain. The chosen attacks and defenses will be performed on the Covid-Net [18] model. The model classifies chest X-ray images into 3 classes (Covid-19, No Disease, and Pneumonia). It forms a good candidate since it achieves a high accuracy of 92.6% and has a different architecture compared to the VGG19 surrogate model.

## 3 Adversarial attack generation framework

The Covid-19 classifier can generate four different outcomes namely, True Positive, True Negative, False Positive, and False Negative. Any trained classifier will have inconsistencies with another trained classifier who has complete authority on a subject, which we refer to as ‘Oracle’. Humans, for example, can act as Oracles in a lot of circumstances. An attacker might take advantage of these inconsistencies to cause the trained classifier to make more errors. This section describes the crafting of adversarial samples by the adversary. As referred to in the paper [19], the adversarial sample generator framework consists of two major operations:

a. Directional sensitivity estimation
b. Perturbation selection

**Figure 1:**
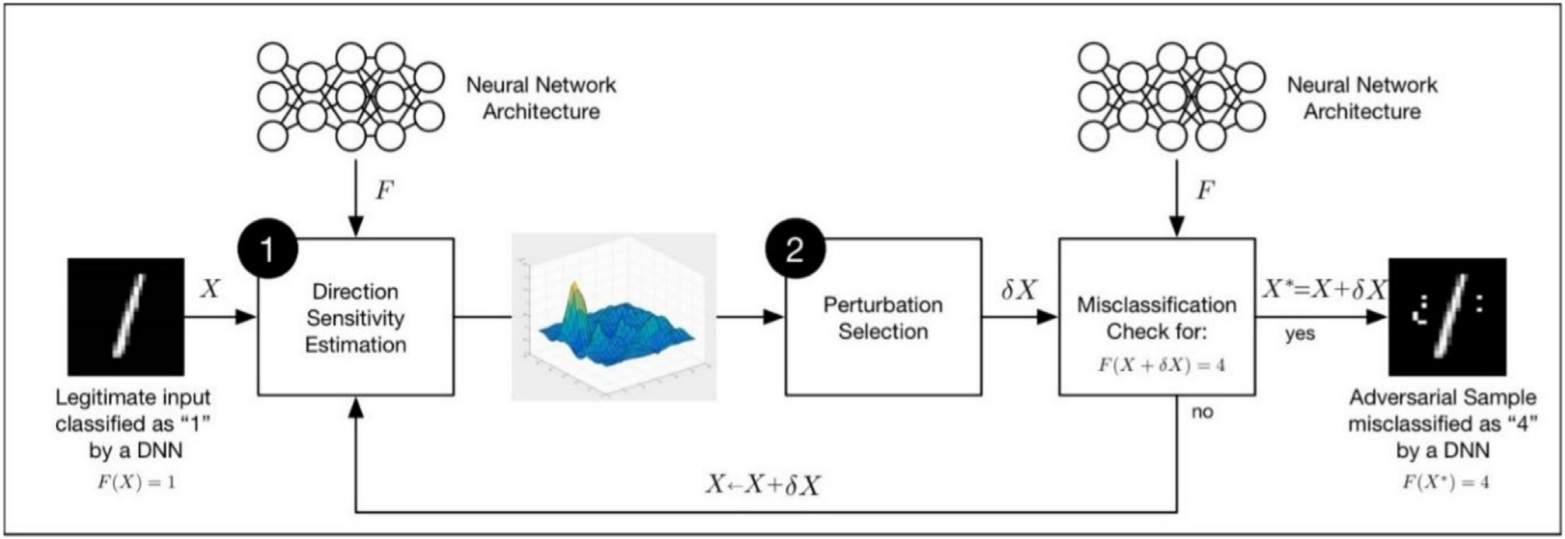
Adversarial example crafting framework [20].

The entire framework is diagrammatically represented in Figure 1. The figure highlights the process of generation of adversarial samples for image classification Deep learning models, which primarily consist of the above-mentioned operations. The framework is for generation of adversarial samples in white box setting. Going into the relevant details of the framework:

Consider a Deep Neural Network F which has been trained on a dataset D. Let θ be parameters of the model. Since a white-box setting is being used, the ability of the adversary to generate adversarial samples is limited by θ. Let X be a clean image sampled from the dataset D such that, F(X) = C_1_, where C_1_ belongs to C, where C is the set of class outputs. The prime objective of the adversary is to generate an adversarial sample X_adv_ from clean sample X (i.e.) X_adv_ = X + δ X such that, F (X_adv_) = $C_{*}$, where $C_{*}$ ≠ C_1_. The output of F when adversarial samples are sent as input solely depends on the objective of the adversary. To achieve this, the adversary begins with the clean sample X and the two processes mentioned above:

a) Directional sensitivity estimation:

This is an evaluation process where the adversary estimates the sensitivity of class change based on the features of the input. This is done by identifying the data regions around the clean image sample in the image space such that the trained model F is sensitive to and is likely to misclassify.

b) Perturbation selection:

The above sensitivity information of the model is then exploited to select the most efficient noise δX such that when added to clean image X, the model D gives out a wrong prediction. However, the degree of perturbation δX must be controlled, i.e., the noise to be added must not be detected by the human eye. Hence, the noise to be added must be as minimum as possible and must also serve its purpose to fool Deep Learning models. In order to achieve this, a norm ||.|| is defined to measure the variance between two samples in the input domain. The generation of adversarial samples based on the above constraint can be treated as solutions to the optimization problem, which can be represented as given in Eq. (1).

(1) $$X_{\text{adv}}=X+\text{argmin}\delta X\left(\left\|\delta X\right\|:F\left(X+\delta X\right)\neq F\left(X\right)\right)$$

The various techniques to generate adversarial samples tend to approximate the solution to the above optimization problem and generate an adversarial sample. The current techniques start off by trying to identify the directional sensitivity of the model. They start with an input sample X and tend to identify the dimensions in the input domain such that a small perturbation added in those dimensions will result in an adversarial sample. The identification of model sensitivity is performed differently by different adversarial generation algorithms. FGSM and PGD are the adversarial attacks that have been used to benchmark the defense in this paper.

### 3.1 Fast Gradient Sign Method (FGSM)

In training a neural network, the updating of weights involves choosing a new set of weights which decreases the loss of the model. This is done by calculating the gradient of the loss with respect to the weights of the model. Since the gradient gives the direction where the loss is maximized, the weights are moved in the opposite direction to minimize the loss at each step. The FGSM attack uses a similar principle to perform the attack. To perform a misclassification, the attack aims to maximize the loss by calculating the gradient of the loss with respect to the input parameters which yields the direction in which the image should be changed to maximize the loss. This is expressed as presented in Eq. (2).

(2) $$x^{\prime }=x+\varepsilon .\text{sign}\left(\nabla_{x}J\left(\Theta ,x,y\right)\right)$$

where,

x′ is the resulting adversarial sample,
x is the original sample,
ε parameter controls the amount of noise added to the original sample.
y is the output of the model
Θ represents the model parameters
▽_x_ J (Θ, x, y) is the gradient of loss w.r.t to x

Since this attack strategy requires access to the model, this kind of an attack is a white-box attack. This process of generating adversarial samples is a one-step process.

### 3.2 Projected Gradient Descent (PGD)

The PGD attack is an iterative white-box attack. This attack attempts to maximize the loss in a number of iterations and hence produces samples that are more likely to be misclassified by the model. To control the amount of noise added to the image, a constraint such as the L∞ norm is specified. Whenever the noise added violates the constraint, the noise is projected back to the norm and the process continues. Since the method tries to maximize the loss iteratively, it is a much stronger attack. On the other hand, it is computationally costly to generate Adversarial samples. The formula for PGD is given by,

(3) $$x_{t+1}^{\prime }=\Pi \left(x_{t}^{\prime }+\varepsilon .\text{sign}\left(\nabla_{x}J\left(\Theta ,x^{\prime },y\right)\right)\right)$$

where,

Π takes the projection in the specified norm.
$x_{t}^{\prime }$ is the adversarial image generated at iteration t
ε parameter controls the amount of noise added to the original sample
y is the output of the model
Θ represents the model parameters
▽_x_ J (Θ, $x_{t}^{\prime }$, y) is the gradient of loss w.r.t to $x_{t}^{\prime }$

## 4 Adversarial defense framework

While discussing potential defenses against adversarial samples, adversarial training is the first explored mechanism. Goodfellow et al. [12], describe adversarial training in detail. Adversarial training involves training the target deep learning model by introducing adversarial samples in the training dataset and generating new adversarial samples in each step of training with that state of the deep learning model at a particular step. This increases the variance of the dataset and better tuning of the model weights so that the degree of misclassification of adversarial samples decreases. This leads to a better generalization of the model. However, in doing so, there is a possibility that it decreases the overall accuracy of the model.

Since an adversarial sample is created by adding controlled perturbation (adversarial noise) to an existing clean sample, the use of Denoisers as a defense can be considered to make the model more robust. The use of denoisers constitutes preprocessing based methods for defense against adversarial samples. The adversarial samples are initially sent as input to a filter which removes the adversarial effect of the added adversarial perturbation. This preprocessed image is sent as input to the image classification model. The denoiser based defense strategy used here, in experiments, called High-Level Representation Guided Denoiser, has been referenced from Fangzhou Liao et al [17].

### 4.1 High-Level Representation Guided Denoiser

The denoiser proposed by Fangzhou Liao et al. [17]. is different from a trivial denoiser present today. Denoising autoencoders (DAE) are some of the most popular denoising models. A DAE consists of an autoencoder network. An autoencoder network is a multilayer perceptron that consists of two major segments, the encoder and the decoder. The encoder section of the autoencoder reduces the dimension of the image to a lower-dimension vector, known as the code. The decoder section of the autoencoder tries to reconstruct the code back to the input. In a typical denoiser network, updates to weights are based on the L_1_ norm of the clean image and the image generated by the denoiser.

(4) L = ||X − X′||

In Eq. (4) X is the input clean image and X′ is the image generated by the DAE. This loss is known as the pixel loss, as a pixel-to-pixel difference is calculated. Denoisers that tune weights based on the pixel loss are known as pixel-guided denoisers. For the image domain, the encoder and the decoder are composed of CNN layers.

Due to the bottlenecking of the trivial DAEs (pixel-guided denoiser) between the encoder and the decoder in reconstructing the input, i.e., lack of transmission of finer-level features from the encoder to decoder, better autoencoder architectures have been considered. An enhanced autoencoder architecture known as DUNET has been developed. A DUNET overcomes the bottleneck problem of the DAE architecture by having lateral connection between the encoder layer and the corresponding decoder layer. Another key difference between both the architectures is that the DUNET architecture learns the noise to be added to the input rather than recreating the input. The noise generated by the DUNET architecture is then added to the adversarial image to remove the adversarial effect. However, the loss function plays a key role in the denoiser architecture. As described in Fangzhou Liao et al. [17], pixel guided is unable to propagate the loss to the deeper layers in the network due to the nature of the perturbation added. The degree of perturbation added to the image is quite small to be captured by the pixel noise. Fangzhou Liao et al. [17]. proposes a new type of loss function calculation that involves a high-level representation of the image. The refined loss function proposed takes the form as stated in Eq. (5).

(5) L_l_ = ||f_l_(X) - f_l_(X_1_) ||

In Eq. (5), X is the clean image from the dataset. X_1_ is the reconstructed image generated by the denoise when it receives adversarial samples generated from clean image X. f_l_(X) is the high-level representation of the clean image X at layer l of the CNN and the f_l_ (X_1_) is the high representation of the image at layer l of reconstructed image X_1_. The layer l used for loss calculation characterizes a different denoiser.

The logit level loss function is calculated as the difference between the logit level representation of X and X_1_. These denoisers are known as Logit-Guided Denoiser (LGD). In our experiments, another loss function which is a combination of both the Pixel Guided Loss (PGL) and Logit Guided Loss (LGL) has been compared. The new loss is specified in Eq. (6).

(6) L = L_1_ + L_2_

The addition of PGL to LGL is based on the hypothesis that PGL will contribute in the tuning of weights of the denoiser so that image generated would be better, getting rid of adversarial noise, and also not distort image generated too much from the clean image.

## 5 Proposed methodology

**Figure 2:**
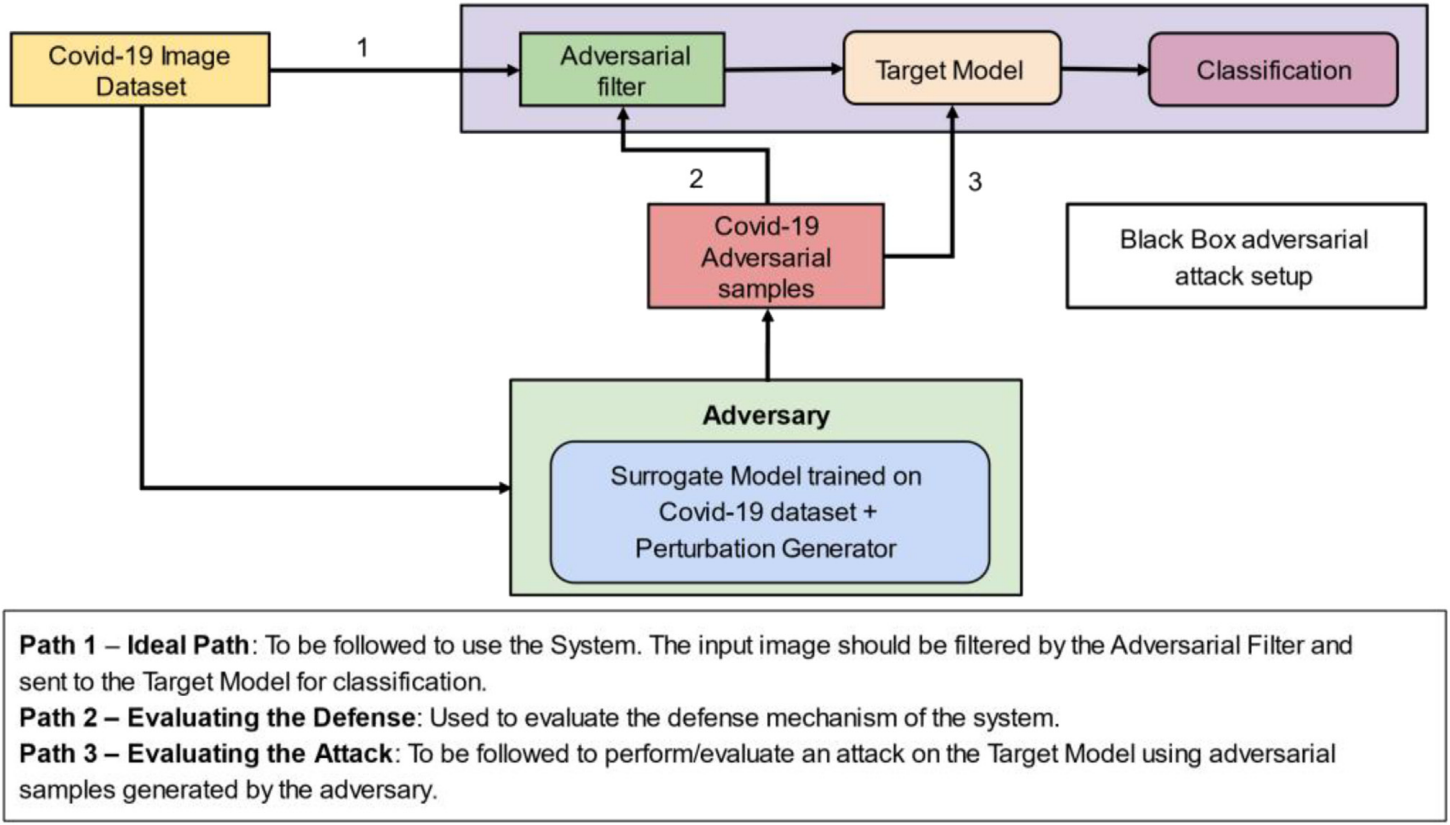
Overview of the proposed system.

This section describes the overall methodology which includes the details of the dataset used in experiments and Surrogate model architecture which is a key component in the Generation of adversarial samples. This is followed by using the adversarial samples to perform attacks and finally the defense methodology. Figure 2 depicts the overall flow of the proposed system.

### 5.1 Dataset

One of the major challenges for Covid-19 classification is the availability of datasets. Since the disease has been recognized much recently, there is scarcity in the number of Covid-19 positive Chest X-Ray images. For all the experiments, images from the CovidX dataset have been used. Compared to other datasets, at the time of this study, this dataset contains the highest number of Covid-19 positive Chest X-Ray samples known at the time of experiments. The dataset is composed of Chest X-Ray images from 3 classes: Normal (No disease), Pneumonia and Covid-19. For training the surrogate model, 4000 images of each class were included. Since the original dataset only included 1600 images labeled as Covid-19 positive, image augmentations such as horizontal flipping, rotation etc. were used to upscale the number to 4000.

### 5.2 Surrogate model

The surrogate model is a model on which the attacker performs the white-box attacks to generate the adversarial samples. It is important to note that the application of this model should be the same as of the target model (such as Covid-19 classification) in order for the adversarial samples to be transferable. This is because the surrogate model functions as an approximation to the target model, irrespective of the architecture or the parameters of the target model.

**Figure 3:**
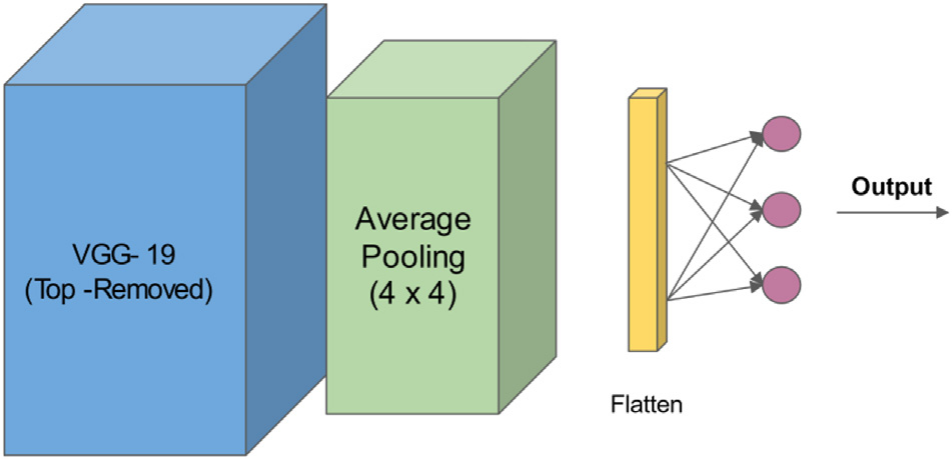
Surrogate model architecture.

For the generation of adversarial samples, a surrogate model for the application of Covid-19 classification is built. We leverage transfer learning and reuse an existing VGG-19 model pre-trained on the ImageNet dataset for the task of Covid-19 classification. Due to the imbalance in the dataset, up-sampling of Covid-19 images was performed by applying data augmentations such as rotation, translation, and horizontal flipping of images. The architecture of the model can be seen in Figure 3.

### 5.3 Generation of adversarial samples

**Figure 4:**
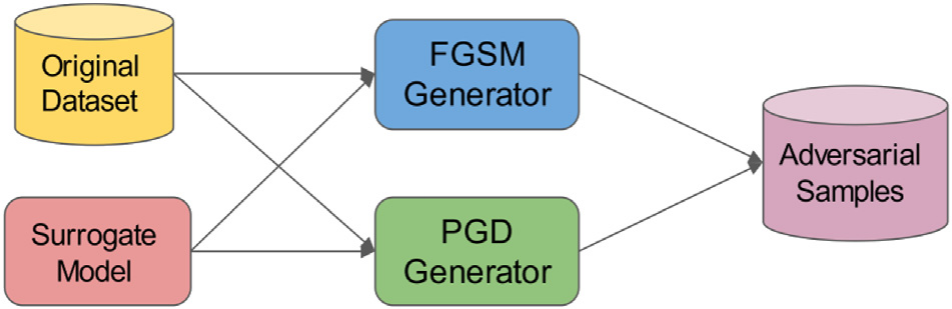
Adversarial sample generation.

The adversarial samples are generated using the two algorithms: FGSM and PGD, taken from the Cleverhans library [21]. A random value for epsilon is chosen from the range of 0.8–8. Each image from the dataset is transformed into an adversarial sample by applying FGSM or PGD. These samples are then sent to the target models for prediction. Figure 4 shows the path taken by a clean image from the dataset to reach the adversarial samples repository. FGSM and PGD generators require a surrogate model as shown in Figure 3. An image is taken from the original dataset and is perturbed by the attacks separately. These generated images form the adversarial samples.

**Figure 5:**
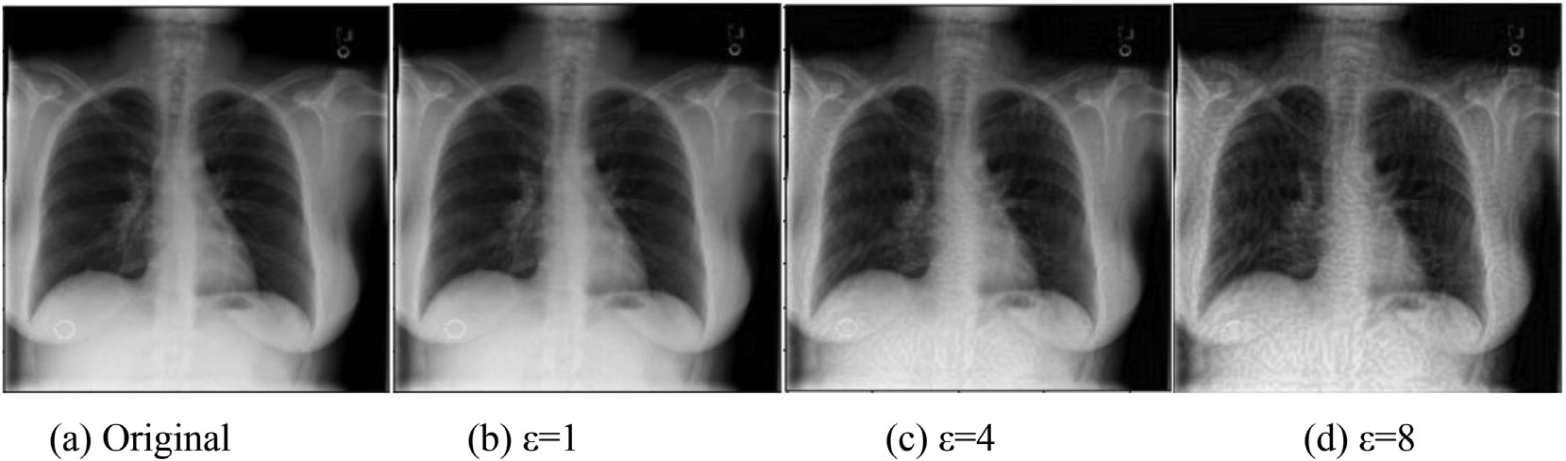
Adversarial Samples Generated by FGSM. (a) Original clean image, (b) Noise factor = 1, (c) Noise factor = 4, (d) Noise factor = 8.

**Figure 6:**
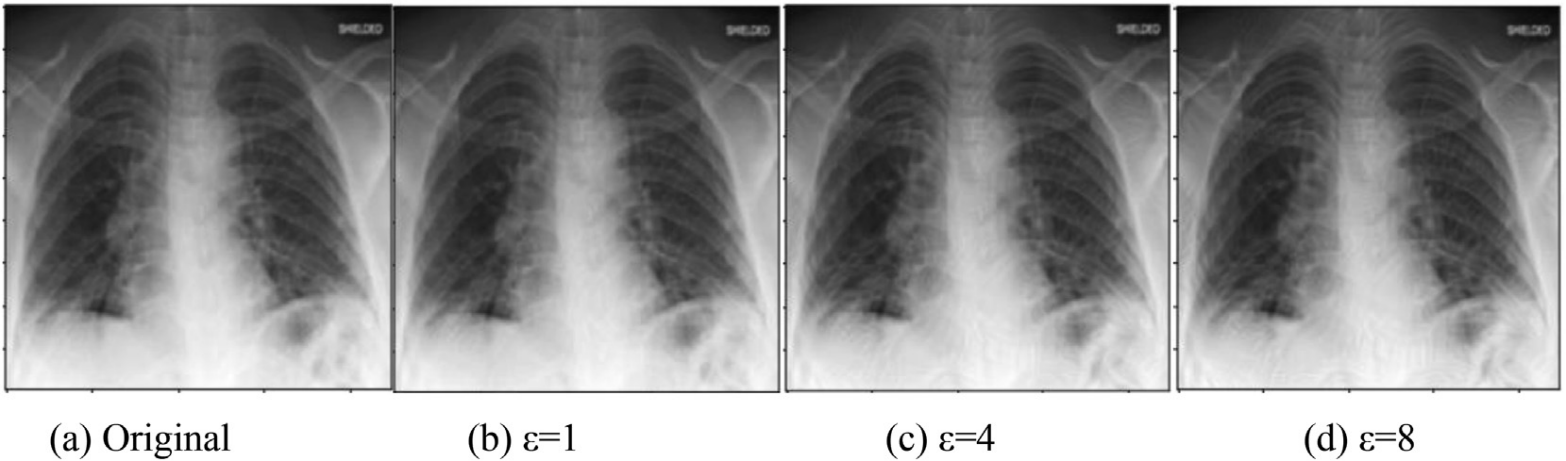
Adversarial Samples Generated by PGD. (a) Original clean image, (b) Noise factor = 1, (c) Noise factor = 4, (d) Noise factor = 8.

**Figure 7:**
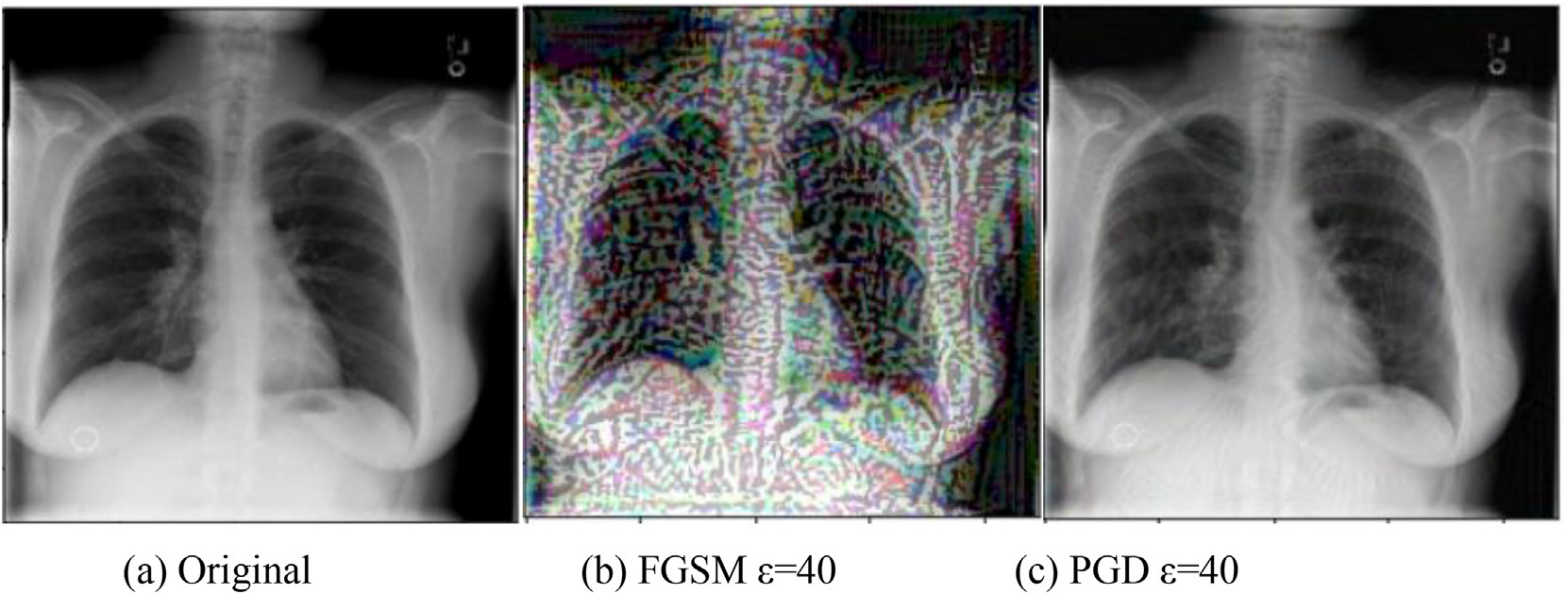
FGSM and PGD Generated Images for High Epsilon Value. (a) Original clean image, (b) Adversarial image generated using FGSM with noise factor = 40, (c) Adversarial image generated using PGD with noise factor = 40.

Figure 5 (a) is an image directly taken from the dataset. FGSM attack is applied on this image with varying values of epsilon as shown in Figure 5 (b–d). The level of visible changes will increase as the value of epsilon increases. Figure 6(a) shows another image taken from the dataset to which PGD attack is applied. The same values of epsilon are taken and are shown in Figures 6 (b–d). It can be observed that PGD generated samples are even more unnoticeable to the naked eye compared to FGSM generated samples. Images given in Figure 7 confirms this with more visibility where high epsilon values are used. Using the original image shown in Figure 7(a), adversarial images are generated using FGSM and PGD methods, shown in Figure 7(b and c) respectively. PGD clearly performs better. It is necessary to strike a trade-off between the distinctly visible perturbations and the model getting fooled. Epsilon is a hyperparameter that needs to be tuned accordingly.

### 5.4 Attack strategy

To perform the classification, traditionally, an image is directly fed to the target model. This leaves the target model vulnerable to adversarial samples. An individual can compromise the results by directly feeding in an adversarial sample or intercepting an image and adding adversarial noise to it. Based on the access to the target model, it can be a white-box attack or a black-box attack.

For the experiments, we consider a scenario where an attacker does not have access to model parameters and architecture, making it a black-box attack. However, to generate adversarial samples using the white-box attacks, another model is created for the same application of Covid-19 classification, called the surrogate model. Adversarial images are generated by performing white-box attacks on the surrogate model and these images are used to test the robustness of the target model and thereby performing a black-box attack on the target model.

### 5.5 Defense strategy

**Figure 8:**
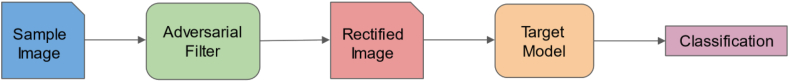
Architecture pipeline.

Defense strategies can be classified as white box and black box. Consider a Denoiser D which is trained using a set of adversarial samples generated using a model A. Let D be used to filter images before it is processed by a Model B for the classification. A defense strategy can be classified as a white box when A = B and as black box when A ≠ B. In order to defend against adversarial samples, the images are passed through a filter before sending them to the target model. The overall flow of a sample image to the classification output is shown in Figure 8. For the experiments, an adversarial filter is trained using the adversarial samples generated using the FGSM and the PGD attack while minimizing the LGD loss which is calculated from the surrogate model.

## 6 Experimental results and discussion

This section describes performance of the surrogate model, performance of both surrogate model and the target model when subjected to adversarial samples in the absence and presence of the defense. The target model chosen here for experiments is the CovidNet Model [18] which has also been trained using the same CovidX dataset as the surrogate model.

### 6.1 Surrogate model performance

The Surrogate Model is validated on the test set (clean samples) included in the CovidX dataset and it achieves an accuracy of **88%**. The Confusion matrix for the surrogate model is shown in Table 1. The diagonal values are high indicating that the model can distinguish between the 3 classes fairly well.

**Table 1:** Confusion matrix for the surrogate model.

True/Predicted	COVID-19	Normal	Pneumonia
COVID-19	82	8	10
Normal	18	832	35
Pneumonia	25	98	471

### 6.2 Attack scenario metrics without defense

The surrogate model and the target model are tested with the image datasets: clean samples and adversarial samples generated by FGSM and PGD. The results in Table 2 show a drastic decrease in the accuracy values when performing attacks in the white box setting. However, in case of the black box setting, the accuracy decrease is not as significant.

**Table 2:** Accuracy before and after respective attacks.

Samples	White Box Setting		Black Box Setting	
	Accuracy	Accuracy Drop	Accuracy	Accuracy Drop
Clean	91%	-	87%	-
FGSM	8%	83%	64%	23%
PGD	8%	83%	67%	20%

The F1 scores shown in Table 3 also confirm this observation. Moreover, it can be seen that the reduction in accuracy when using adversarial samples generated by FGSM is higher compared to the accuracy drop seen with samples generated using the PGD attack method. This is unexpected since the PGD method is assumed to generate samples that are more likely to be misclassified. This suggests that not all the adversarial samples generated using the surrogate model generalize and the noise added by the white-box attacks exploits the vulnerabilities of the model on which the attacks are performed. These vulnerabilities may not be shared by another model for the same application.

**Table 3:** F1 Score after respective attacks.

White Box Setting			
	Covid-19	Normal	Pneumonia
FGSM	0.06	0.04	0.14
PGD	0.03	0.04	0.14

Black Box Setting			
	Covid-19	Normal	Pneumonia
FGSM	0.63	0.68	0.63
PGD	0.65	0.70	0.68

### 6.3 Attack scenario metrics with defense

Further, the different defense architectures are tested with adversarial samples generated by FGSM and PGD. Table 4 shows the accuracies recomputed after using the proposed defense architectures. In the white-box setting, it can be seen that the PGL-driven defense architecture fails to improve the accuracy. As mentioned in section 4, due to the nature of adversarial noise added, the pixel noise fails to tune the weights of the denoiser to remove the effect of the added adversarial noise. Hence, with PGL defense no change in accuracy is observed. In contrast, while using the LGL in training of the denoiser, a noticeable improvement of 74% is observed. The LGL, unlike the PGL, captures the loss between higher-level representation (logit level) of the adversarial image and the clean image, leading to generation of loss that helps in the weight-tuning of layers deeper in the network. This leads to the LGL-driven defense architecture performing much better than the PGL-driven defense architecture. Though the use of LGL is very successful in reducing the nature of adversarial noise from an adversarial image, it cares less about how it generates the adversarial-effect-free image. The PGL + LGL driven architecture increases the accuracy of the model, yet the increase is not as good as the improvement seen in the LGL-driven defense.

**Table 4:** Accuracy with defenses.

Defense Architecture	White Box Setting		Black Box Setting	
	Accuracy	Increase in Accuracy	Accuracy	Increase in Accuracy
PGL	8%	0%	64%	0%
LGL	82%	74%	33%	-31%
PGL + LGL	76%	68%	38%	-26%

However, similar results are not observed when considering a black box setting. The PGL-driven defense architecture sees no improvement in accuracy, which is expected as mentioned previously. The LGL and LGL + PGL driven defenses further degrade the performance of the model. The degrading performance can be traced back to the training of the defense. Since the defense architecture was initially trained on the surrogate model, the defense architecture develops a high dependency on the surrogate model. The F1 scores presented in Table 5 confirms the inadequacy of the defense techniques in a black box setting.

**Table 5:** F1 Score with defenses.

White Box Setting			
	Covid-19	Normal	Pneumonia
PGL	0.05	0.04	0.14
LGL	0.85	0.81	0.79
LGL + PGL	0.81	0.74	0.74

Black Box Setting			
	Covid 19	Normal	Pneumonia
PGL	0.63	0.68	0.63
LGL	0.00	0.50	0.00
LGL + PGL	0.52	0.02	0.28

**Figure 9:**
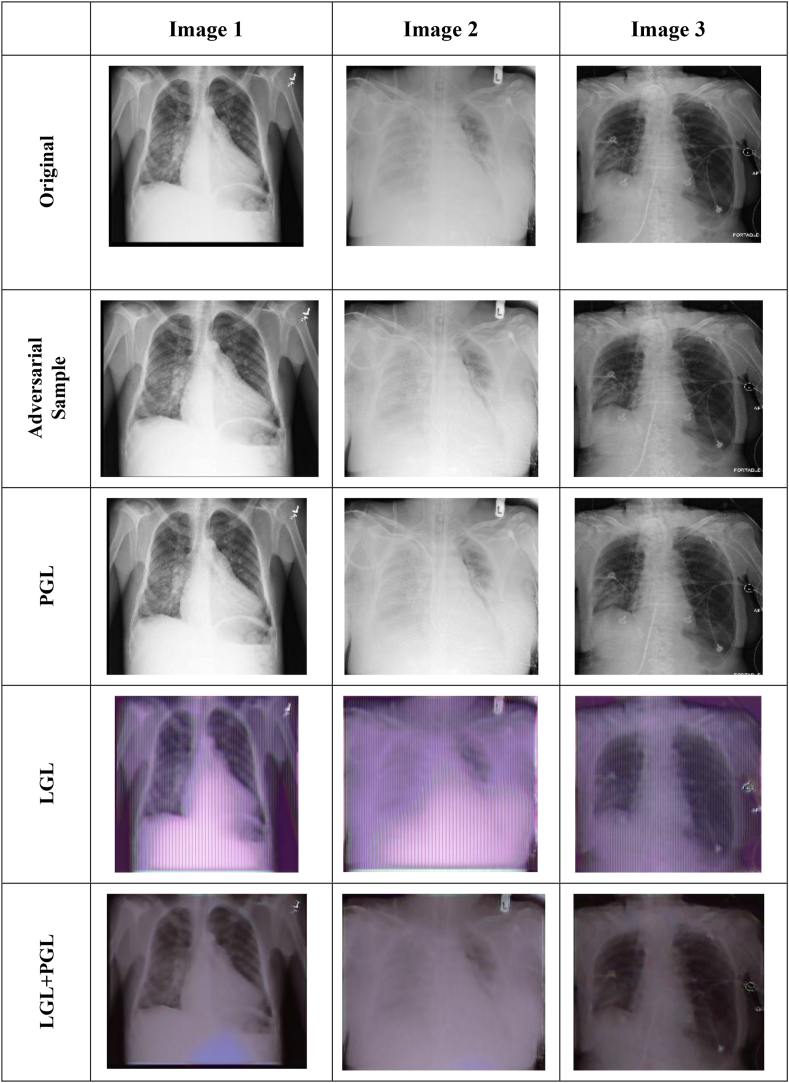
Images generated by the different defense architectures. Row 1 depicts the original clean images, row 2 depicts adversarial images generated using FGSM or PGD methods, rows 3–5 depict images generated by a PGL, LGL, LGL + PGL based denoisers respectively.

All modifications done to the adversarial image to remove the adversarial noise effect are done to increase the performance of the surrogate model. Similar modifications to the adversarial images may not favor the classification function approximated by the target model, and hence the degradation in the accuracy. Figure 9 shows the images generated by each of the defenses. Since the PGL considers the pixel-wise difference in the images it shows visibly no difference from the original image. LGL only considers the loss between the higher layers of the model and the output image is much more distinct from the original. In case of the new loss function proposed i.e., LGL + PGL, the output image is much better than the image generated by LGL.

## 7 Conclusion

The detection of Covid-19 using Chest X-Rays is a significant step towards speeding up the testing process for detection of the virus. Adversarial samples given to such models can compromise results in both the white-box as well as the black-box setting. However, after the addition of an adversarial filter, HGD, in a white box setting, an increase in metrics is observed when using the LGL and the new proposed loss function, LGL + PGL defense. These may lead to undesirable changes to the original image. However, as seen, changes in the image can be mitigated by using different loss functions, which include a combination of pixel and logit level loss. The PGL architecture completely fails to improve the accuracy. In the black box setting, the defense architecture fails and even leads to a decrease in accuracy. This suggests that the HGD defense architecture is a good candidate for a defense strategy in a white box setting as it does not lead to re-training of the target model which is being defended. Results in a black box setting suggest that it is not transferable across models, which may be because of the differences in the performance of the surrogate model and the target model.

## 8 Limitations and future work

At the time of experiments, the major challenge was the unavailability of exhaustive Covid-19 Datasets. Including additional Covid-19 positive images in the dataset will lead to better tuning of surrogate model weights, thereby generating better adversarial samples. The scope of this paper included two attacks, namely FGSM and PGD. However, more effective attacks such as GAN based attacks [22] and ensemble methods [23] can be included in the experiments to prepare a more robust denoiser. With respect to HGD, more loss functions can be explored and included in the study which may lead to a more robust defense.

## Declarations

### Author contribution statement

Keshav Kansal, Sai P Krishna, Surya R: Conceived and designed the experiments; Performed the experiments; Analyzed and interpreted the data; Contributed reagents, materials, analysis tools or data; Wrote the paper.

Parshva B Jain: Conceived and designed the experiments; Performed the experiments; Analyzed and interpreted the data.

Prasad Honnavalli: Conceived and designed the experiments; Contributed reagents, materials, analysis tools or data.

Sivaraman Eswaran: Conceived and designed the experiments; Analyzed and interpreted the data; Wrote the paper.

### Funding statement

This research did not receive any specific grant from funding agencies in the public, commercial, or not-for-profit sectors.

### Data availability statement

Data will be made available on request.

### Declaration of interests statement

The authors declare no conflict of interest.

### Additional information

No additional information is available for this paper.
